# Supplementary material for: Modelling Skylarks (Alauda arvensis) to Predict Impacts of Changes in Land Management and Policy: Development and Testing of an Agent-Based Model
Source: PLoS One. 2013 Jun 6;8(6):e65803. doi: 10.1371/journal.pone.0065803 (PMC3675089; doi:10.1371/journal.pone.0065803)
Supplement: Supporting Information S4 — The skylark ODdox as a zipped archive. (ZIP) [file pone.0065803.s004.zip › Skylark_ODdox/class_skylark___female.html]

ALMaSS Skylark ODdox: Skylark\_Female Class Reference


ALMaSS Skylark ODdox
 2.0


- Main Page
- Related Pages
- Classes
- Files

- Class List
- Class Index
- Class Hierarchy
- Class Members

Public Member Functions |
Public Attributes |
Protected Member Functions |
Protected Attributes

Skylark\_Female Class Reference

`#include <skylarks_all.h>`

List of all members.

|  |  |
| --- | --- |
| Public Member Functions | |
| virtual void | BeginStep (void) |
| virtual void | EndStep (void) |
| void | EstablishTerritory () |
| void | OnBreedingSuccess () |
| void | OnBreedSuccess () |
| void | OnBroodDeath () |
| void | OnClutchDeath () |
| void | OnEggsHatch () |
| void | OnMaleNeverComesBack (Skylark\_Male \*AMale) |
| void | OnMateDying () |
| void | OnMateHomeless () |
| void | OnSetMyClutch (Skylark\_Clutch \*p\_C) |
| void | OnStopFeedingChicks () |
| void | ResetBreedingSuccess () |
| void | SensibleCopy () |
|  | Skylark\_Female (int x, int y, double size, int age, SkTerritories \*Terrs, Landscape \*L, Skylark\_Population\_Manager \*SPM, int bx, int by, int mh) |
| virtual void | Step (void) |
| int | Supply\_BreedingAttempts () |
| int | Supply\_BreedingSuccess () |
| int | Supply\_NestTime () |
| Skylark\_Clutch \* | SupplyMyClutch () |
| Public Member Functions inherited from Skylark\_Adult | |
| virtual void | CopyMyself (int a\_sktype) |
|  | Skylark\_Adult (int x, int y, double size, int age, SkTerritories \*Terrs, Landscape \*L, Skylark\_Population\_Manager \*SPM, int bx, int by, int mh) |
|  | ~Skylark\_Adult () |
| Public Member Functions inherited from Skylark\_Base | |
| void | AddStriglingMort (int lifestage) |
| bool | InSquare (rectangle R) |
| virtual double | On\_FoodSupply (double) |
|  | Skylark\_Base (int x, int y, SkTerritories \*Terrs, Landscape \*L, Skylark\_Population\_Manager \*SPM, int bx, int by, int mh) |
| virtual int | WhatState () |
| Public Member Functions inherited from TAnimal | |
| void | CheckManagement (void) |
| void | CheckManagementXY (int x, int y) |
| virtual void | CopyMyself () |
| virtual void | Dying () |
| virtual void | KillThis () |
| void | SetX (int a\_x) |
| void | SetY (int a\_y) |
| int | Supply\_m\_Location\_x () |
| int | Supply\_m\_Location\_y () |
| unsigned | SupplyFarmOwnerRef () |
| int | SupplyPolygonRef () |
| AnimalPosition | SupplyPosition () |
|  | TAnimal (int x, int y, Landscape \*L) |
| Public Member Functions inherited from TALMaSSObject | |
| void | OnArrayBoundsError () |
|  | TALMaSSObject () |
| virtual | ~TALMaSSObject () |

|  |  |
| --- | --- |
| Public Attributes | |
| int | m\_CIPEGrid [2][6] |
| Skylark\_Male \* | MyMate |
| Public Attributes inherited from Skylark\_Adult | |
| bool | Paired |
| Public Attributes inherited from Skylark\_Base | |
| int | Age |
| int | m\_Born\_x |
| int | m\_Born\_y |
| TTypesOfSkState | m\_CurrentSkState |
| int | m\_MyHome |
|  | The vegetation type where the skylark was born. |
| Skylark\_Population\_Manager \* | m\_OurPopulationManager |
| SkTerritories \* | m\_OurTerritories |
| double | m\_pcide\_conc |
| double | m\_pesticide\_accumulation |
| double | m\_Size |

|  |  |
| --- | --- |
| Protected Member Functions | |
| int | CalcFoodTime (double target) |
| int | CalculateEggNumber () |
| double | CheckForFields () |
| void | FeedYoung () |
| double | GetFood (int time) |
| int | GetMigrationMortality () |
| virtual bool | OnFarmEvent (FarmToDo event) |
| virtual void | PesticideResponse () |
| skSpiralResult | Spiral (int x, int y, int radius) |
| skSpiralResult | Spiral2 (int x, int y, int radius) |
| bool | st\_Arriving () |
| int | st\_BuildingUpResources () |
| int | st\_CaringForYoung () |
| void | st\_Dying () |
| int | st\_EggHatching () |
| int | st\_Emigrating () |
| int | st\_Finding\_Territory () |
| int | st\_Floating () |
| int | st\_Flocking () |
| int | st\_GivingUpTerritory () |
| bool | st\_Immigrating () |
| int | st\_Incubating () |
| int | st\_Laying () |
| int | st\_MakingNest () |
| int | st\_PreparingForBreeding () |
| int | st\_StartingNewBrood () |
| int | st\_StoppingBreeding () |
| int | st\_TempLeavingArea () |
| Protected Member Functions inherited from Skylark\_Adult | |
| bool | GetBadWeather () |
|  | Extreme weather conditions check. |
| double | GetVegHindrance (int PolyRef) |
| double | GetWeatherHindrance () |
| virtual double | RemoveEM (double food) |
| Protected Member Functions inherited from Skylark\_Base | |
| bool | DailyMortality (int mort) |
| Protected Member Functions inherited from TAnimal | |
| void | CorrectWrapRound (void) |
|  | Corrects wrap around co-ordinate problems. |

|  |  |
| --- | --- |
| Protected Attributes | |
| int | EggCounter |
| int | m\_BreedingAttempts |
| int | m\_BreedingSuccess |
| int | m\_Counter1 |
| int | m\_EggNumber |
| double | m\_MinFemaleAcceptScore |
| int | m\_NestTime |
| bool | m\_pesticide\_sprayed |
| bool | m\_pesticide\_sprayed\_die |
| int | m\_toowet |
| Skylark\_Clutch \* | MyClutch |
| bool | NestLoc |
| double | Resources |
| Protected Attributes inherited from Skylark\_Adult | |
| bool | BSuccess |
| int | GoodWeather |
| vector< Point > \* | m\_aTerrlist |
| double | MyExtractEff |
| skTerritory\_struct | MyTerritory |

---

## Constructor & Destructor Documentation

|  |  |  |  |
| --- | --- | --- | --- |
| Skylark\_Female::Skylark\_Female | ( | int | *x*, |
|  |  | int | *y*, |
|  |  | double | *size*, |
|  |  | int | *age*, |
|  |  | SkTerritories \* | *Terrs*, |
|  |  | Landscape \* | *L*, |
|  |  | Skylark\_Population\_Manager \* | *SPM*, |
|  |  | int | *bx*, |
|  |  | int | *by*, |
|  |  | int | *mh* |
|  | ) |  |  |

References m\_BreedingAttempts, m\_MinFemaleAcceptScore, m\_pesticide\_sprayed, MyClutch, MyMate, and ResetBreedingSuccess().

: Skylark\_Adult( x, y, size, age, Terrs, L, SPM, bx, by, mh ) {

MyMate = NULL;

MyClutch = NULL;

ResetBreedingSuccess();

m\_BreedingAttempts = 0;

m\_pesticide\_sprayed = false;

// m\_pesticide\_sprayed\_die=false;

// Incorporate 10% variation in quality acceptance

m\_MinFemaleAcceptScore = 0;

}

---

## Member Function Documentation

|  |  |  |  |  |  |
| --- | --- | --- | --- | --- | --- |
| void Skylark\_Female::BeginStep | ( | void |  | ) |  |

virtual

Reimplemented from TAnimal.

References TAnimal::CheckManagement(), Skylark\_Base::m\_CurrentSkState, m\_MinFemaleAcceptScore, st\_CaringForYoung(), st\_Floating(), TALMaSSObject::StepDone, toss\_Destroy, toss\_FCaringForYoung, toss\_FFindingTerritory, toss\_FFloating, toss\_FFlocking, toss\_FTempLeavingArea, toss\_StartingNewBrood, and toss\_StoppingBreeding.

{

#ifdef \_\_CJTDebug\_5

if ( IsAlive() != 0x0DEADC0DE ) DEADCODEError();

#endif

if ( m\_CurrentSkState == toss\_Destroy ) return;

CheckManagement();

if (m\_MinFemaleAcceptScore<0.0) m\_MinFemaleAcceptScore = 0.0;

switch ( m\_CurrentSkState ) {

case toss\_FFloating: // Floating

switch ( st\_Floating() ) {

case 2:

m\_CurrentSkState = toss\_FFlocking;

break;

case 3:

m\_CurrentSkState = toss\_FTempLeavingArea; // -> temp.leave

break;

case 1:

m\_CurrentSkState = toss\_FFindingTerritory; // -> find terr.

break;

case 0:

break;

}

StepDone = true;

break;

case toss\_FCaringForYoung: // CaringForYoung

switch ( st\_CaringForYoung() ) {

case 2: //StopBreeding

m\_CurrentSkState = toss\_StoppingBreeding;

break;

case 1:

m\_CurrentSkState = toss\_StartingNewBrood;

break;

default:

break;

}

StepDone = true;

break;

default:

/\* char errornum[20]; sprintf(errornum, "%d", m\_CurrentSkState );

g\_land->Warn("Skylark\_Female::BeginStep(): Unknown state: ", errornum); exit(0); \*/

break;

}

}

|  |  |  |  |  |  |
| --- | --- | --- | --- | --- | --- |
| int Skylark\_Female::CalcFoodTime | ( | double | *target* | ) |  |

protected

Referenced by st\_Incubating().

|  |  |  |  |  |
| --- | --- | --- | --- | --- |
| int Skylark\_Female::CalculateEggNumber | ( |  | ) |  |

protected

Referenced by st\_Laying().

|  |  |  |  |  |
| --- | --- | --- | --- | --- |
| double Skylark\_Female::CheckForFields | ( |  | ) |  |

protected

|  |  |  |  |  |  |
| --- | --- | --- | --- | --- | --- |
| void Skylark\_Female::EndStep | ( | void |  | ) |  |

virtual

Reimplemented from TAnimal.

References PesticideResponse().

{

#ifdef \_\_CJTDebug\_5

if ( IsAlive() != 0x0DEADC0DE ) DEADCODEError();

#endif

// Need to find out if it is poisoned

#ifdef \_\_PESTICIDE\_RA

PesticideResponse();

#endif

}

|  |  |  |  |  |
| --- | --- | --- | --- | --- |
| void Skylark\_Female::EstablishTerritory | ( |  | ) |  |

References Skylark\_Adult::BSuccess, SkTerritories::FemaleOccupy(), TAnimal::m\_Location\_x, TAnimal::m\_Location\_y, Skylark\_Base::m\_OurPopulationManager, Skylark\_Base::m\_OurTerritories, MyMate, Skylark\_Adult::MyTerritory, Skylark\_Male::OnPairing(), Skylark\_Adult::Paired, skTerritory\_struct::ref, Resources, Population\_Manager::SimH, Population\_Manager::SimW, skTerritory\_struct::size, Skylark\_Male::Supply\_Territory(), skTerritory\_struct::x, and skTerritory\_struct::y.

Referenced by Skylark\_Male::OnReHouse(), and st\_Finding\_Territory().

{

#ifdef \_\_CJTDebug\_5

if ( IsAlive() != 0x0DEADC0DE ) DEADCODEError();

#endif

MyMate->OnPairing( this );

Paired = true;

MyTerritory = MyMate->Supply\_Territory();

Resources = 0;

// Move 31-01-2007 to Immigrating m\_BreedingAttempts = 0;

m\_OurTerritories->FemaleOccupy( MyTerritory.ref, this );

m\_Location\_x = ( ( MyTerritory.size / 2 ) + MyTerritory.x + 5 ) % m\_OurPopulationManager->SimW; // next to male

m\_Location\_y = ( ( MyTerritory.size / 2 ) + MyTerritory.y ) % m\_OurPopulationManager->SimH;

BSuccess = false; // reset the breeding success flag

}

|  |  |  |  |  |
| --- | --- | --- | --- | --- |
| void Skylark\_Female::FeedYoung | ( |  | ) |  |

protected

Can only get food in daylight - dawn & dusk. Must keep them warm if the weather is bad - the quick version below catagorizes this into good or bad weather

References FoodTripsPerDay, GetFood(), m\_NestTime, TAnimal::m\_OurLandscape, Skylark\_Base::m\_pcide\_conc, MaxFeedRain, MyMate, Skylark\_Male::OnFoodMessage(), random(), Skylark\_Adult::RemoveEM(), Skylark\_Male::SupplyBroodAge(), Skylark\_Male::SupplyBroodSize(), Skylark\_Male::SupplyBroodWeight(), Landscape::SupplyDaylength(), Landscape::SupplyRain(), and Landscape::SupplyTemp().

Referenced by st\_CaringForYoung(), and st\_GivingUpTerritory().

|  |  |  |  |  |  |
| --- | --- | --- | --- | --- | --- |
| double Skylark\_Female::GetFood | ( | int | *time* | ) |  |

protected

Referenced by FeedYoung(), and st\_PreparingForBreeding().

|  |  |  |  |  |
| --- | --- | --- | --- | --- |
| int Skylark\_Female::GetMigrationMortality | ( |  | ) |  |

protected

Referenced by st\_Immigrating().

|  |  |  |  |  |
| --- | --- | --- | --- | --- |
| void Skylark\_Female::OnBreedingSuccess | ( |  | ) |  |

References Skylark\_Adult::BSuccess.

Referenced by Skylark\_Male::OnPreFledgelingMature(), and Skylark\_Male::st\_ScaringOffChicks().

{

#ifdef \_\_CJTDebug\_5

if ( IsAlive() != 0x0DEADC0DE ) DEADCODEError();

#endif

BSuccess = true;

}

|  |  |  |  |  |
| --- | --- | --- | --- | --- |
| void Skylark\_Female::OnBreedSuccess | ( |  | ) |  |

inline

Referenced by Skylark\_Male::OnAddPreFledgeling().

{ m\_BreedingSuccess++; }

|  |  |  |  |  |
| --- | --- | --- | --- | --- |
| void Skylark\_Female::OnBroodDeath | ( |  | ) |  |

References m\_Counter1, Skylark\_Base::m\_CurrentSkState, MyClutch, Skylark\_Adult::Paired, and toss\_StartingNewBrood.

Referenced by Skylark\_Male::OnBroodDeath().

{

#ifdef \_\_CJTDebug\_5

if ( IsAlive() != 0xDEADC0DE )

DEADCODEError();

#endif

if ( Paired ) // if !Paired then ignore them, must have deserted

{

// Start a new brood if not already done so naturally

if ( !MyClutch ) {

m\_Counter1 = 5;

m\_CurrentSkState = toss\_StartingNewBrood;

}

}

}

|  |  |  |  |  |
| --- | --- | --- | --- | --- |
| void Skylark\_Female::OnClutchDeath | ( |  | ) |  |

References m\_Counter1, Skylark\_Base::m\_CurrentSkState, MyClutch, Skylark\_Adult::Paired, toss\_StartingNewBrood, and Landscape::Warn().

Referenced by Skylark\_Clutch::st\_Dying().

{

#ifdef \_\_CJTDebug\_5

if ( IsAlive() != 0xDEADC0DE )

DEADCODEError();

#endif

if ( Paired ) // if !Paired then ignore the message, must have deserted

{

#ifdef \_\_CJTDebug\_7

if ( !MyClutch ) {

g\_land->Warn( "Skylark\_Female::OnClutchDeat - No clutch to die ", NULL );

exit( 1 );

}

#endif

MyClutch = NULL;

m\_Counter1 = 5;

m\_CurrentSkState = toss\_StartingNewBrood; //Go to new brood start

}

}

|  |  |  |  |  |
| --- | --- | --- | --- | --- |
| void Skylark\_Female::OnEggsHatch | ( |  | ) |  |

References Skylark\_Base::m\_CurrentSkState, MyClutch, MyMate, Skylark\_Male::OnEggHatch(), toss\_EggHatching, and Landscape::Warn().

Referenced by Skylark\_Clutch::st\_Hatching().

{

#ifdef \_\_CJTDebug\_5

if ( IsAlive() != 0xDEADC0DE )

DEADCODEError();

#endif

// This function must not be called until the nestling objects have been

// created

#ifdef \_\_CJTDebug\_7

if ( !MyClutch ) {

g\_land->Warn( "Skylark\_Female::OnEggsHatch - No clutch to hatch ", NULL );

exit( 1 );

}

#endif

MyClutch = NULL;

m\_CurrentSkState = toss\_EggHatching; //Go to EggHatching

// Tell the male

MyMate->OnEggHatch();

}

|  |  |  |  |  |  |
| --- | --- | --- | --- | --- | --- |
| bool Skylark\_Female::OnFarmEvent | ( | FarmToDo | *event* | ) |  |

protectedvirtual

Reimplemented from TAnimal.

References Skylark\_Base::AddStriglingMort(), autumn\_harrow, autumn\_or\_spring\_plough, autumn\_plough, autumn\_roll, autumn\_sow, burn\_straw\_stubble, cattle\_out, cattle\_out\_low, cut\_to\_hay, cut\_to\_silage, cut\_weeds, Calendar::Date(), deep\_ploughing, Landscape::EventtypeToString(), fa\_ammoniumsulphate, fa\_greenmanure, fa\_manure, fa\_npk, fa\_pk, fa\_sludge, fa\_slurry, fp\_greenmanure, fp\_liquidNH3, fp\_manganesesulphate, fp\_manure, fp\_npk, fp\_npks, fp\_pk, fp\_sludge, fp\_slurry, fungicide\_treat, g\_date, glyphosate, growth\_regulator, harvest, hay\_bailing, hay\_turning, herbicide\_treat, hilling\_up, insecticide\_treat, Skylark\_Base::m\_CurrentSkState, TAnimal::m\_OurLandscape, m\_pesticide\_sprayed, molluscicide, mow, Skylark\_Adult::Paired, pigs\_out, product\_treat, random(), row\_cultivation, sleep\_all\_day, spring\_harrow, spring\_plough, spring\_roll, spring\_sow, straw\_chopping, strigling, strigling\_sow, stubble\_harrowing, swathing, syninsecticide\_treat, toss\_FDying, toss\_GivingUpTerritory, toss\_MakingNest, toss\_PreparingForBreeding, toss\_StartingNewBrood, trial\_insecticidetreat, CfgInt::value(), Landscape::Warn(), water, and winter\_plough.

{

switch ( event ) {

case sleep\_all\_day:

break;

case autumn\_plough:

// If she is in nest building then the nest is destroyed

if (( m\_CurrentSkState == toss\_MakingNest) || (m\_CurrentSkState == toss\_PreparingForBreeding)) {

// Make a state change

m\_CurrentSkState = toss\_StartingNewBrood;

}

break;

case autumn\_harrow:

// If she is in nest building then the nest is destroyed

if (( m\_CurrentSkState == toss\_MakingNest) || (m\_CurrentSkState == toss\_PreparingForBreeding)) {

// Make a state change

m\_CurrentSkState = toss\_StartingNewBrood;

}

break;

case autumn\_roll:

break;

case autumn\_sow:

// If she is in nest building then the nest is destroyed

if (( m\_CurrentSkState == toss\_MakingNest) || (m\_CurrentSkState == toss\_PreparingForBreeding)) {

// Make a state change

m\_CurrentSkState = toss\_StartingNewBrood;

}

break;

case winter\_plough:

// If she is in nest building then the nest is destroyed

if (( m\_CurrentSkState == toss\_MakingNest) || (m\_CurrentSkState == toss\_PreparingForBreeding)) {

// Make a state change

m\_CurrentSkState = toss\_StartingNewBrood;

}

break;

case deep\_ploughing:

// If she is in nest building then the nest is destroyed

if (( m\_CurrentSkState == toss\_MakingNest) || (m\_CurrentSkState == toss\_PreparingForBreeding)) {

// Make a state change

m\_CurrentSkState = toss\_StartingNewBrood;

}

break;

case spring\_plough:

// If she is in nest building then the nest is destroyed

if (( m\_CurrentSkState == toss\_MakingNest) || (m\_CurrentSkState == toss\_PreparingForBreeding)) {

// Make a state change

m\_CurrentSkState = toss\_StartingNewBrood;

}

break;

case spring\_harrow:

// If she is in nest building then the nest is destroyed

if (( m\_CurrentSkState == toss\_MakingNest) || (m\_CurrentSkState == toss\_PreparingForBreeding)) {

// Make a state change

m\_CurrentSkState = toss\_StartingNewBrood;

}

break;

case spring\_roll:

break;

case spring\_sow:

// If she is in nest building then the nest is destroyed

if (( m\_CurrentSkState == toss\_MakingNest) || (m\_CurrentSkState == toss\_PreparingForBreeding)) {

// Make a state change

m\_CurrentSkState = toss\_StartingNewBrood;

}

break;

case fp\_npks:

break;

case fp\_npk:

break;

case fp\_pk:

break;

case fp\_liquidNH3:

break;

case fp\_slurry:

break;

case fp\_manganesesulphate:

break;

case fp\_manure:

break;

case fp\_greenmanure:

break;

case fp\_sludge:

break;

case fa\_npk:

break;

case fa\_pk:

break;

case fa\_slurry:

break;

case fa\_ammoniumsulphate:

break;

case fa\_manure:

break;

case fa\_greenmanure:

break;

case fa\_sludge:

break;

case herbicide\_treat:

break;

case growth\_regulator:

break;

case fungicide\_treat:

break;

case insecticide\_treat:

case trial\_insecticidetreat:

break;

case syninsecticide\_treat:

if ( random( 1000 ) < cfg\_insecticide\_direct\_mortF.value() ) {

#ifdef TEST\_ISSUE\_DEATH\_WARRANT

printf( "Skylark\_Female::OnFarmEvent() : %d : %d\n", event, ( int )g\_date->Date() );

#endif

m\_CurrentSkState = toss\_FDying;

}

m\_pesticide\_sprayed = true;

break;

case molluscicide:

break;

case row\_cultivation:

// If she is in nest building then the nest is destroyed

if (( m\_CurrentSkState == toss\_MakingNest) || (m\_CurrentSkState == toss\_PreparingForBreeding)) {

// Make a state change

m\_CurrentSkState = toss\_StartingNewBrood;

}

break;

case strigling:

// If she is in nest building then the nest is destroyed

if ( m\_CurrentSkState == toss\_MakingNest ) {

// Make a state change

m\_CurrentSkState = toss\_StartingNewBrood;

AddStriglingMort( 0 );

}

break;

case hilling\_up:

break;

case water:

break;

case swathing:

break;

case harvest:

break;

case cattle\_out:

case cattle\_out\_low:

break;

case cut\_to\_hay:

break;

case cut\_to\_silage:

break;

case straw\_chopping:

break;

case hay\_turning:

break;

case hay\_bailing:

break;

case stubble\_harrowing:

// If she is in nest building then the nest is destroyed

if ( m\_CurrentSkState == toss\_MakingNest ) {

// Make a state change

m\_CurrentSkState = toss\_StartingNewBrood;

}

break;

case autumn\_or\_spring\_plough:

// If she is in nest building then the nest is destroyed

if (( m\_CurrentSkState == toss\_MakingNest) || (m\_CurrentSkState == toss\_PreparingForBreeding)) {

// Make a state change

m\_CurrentSkState = toss\_StartingNewBrood;

}

break;

case burn\_straw\_stubble:

break;

case mow:

break;

case cut\_weeds:

break;

case pigs\_out:

#ifndef \_\_NoPigsOutEffect

if ( Paired ) {

if ( random( 100 ) < 2 ) // 2%

{

// Too much disturbance so give up

m\_CurrentSkState = toss\_GivingUpTerritory;

}

}

#endif

break;

case strigling\_sow:

// If she is in nest building then the nest is destroyed

if (( m\_CurrentSkState == toss\_MakingNest) || (m\_CurrentSkState == toss\_PreparingForBreeding)) {

// Make a state change

m\_CurrentSkState = toss\_StartingNewBrood;

AddStriglingMort( 0 );

}

break;

case product\_treat:

break;

case glyphosate:

break;

default:

g\_land->Warn( "Skylark\_Female::OnFarmEvent(): ""Unknown event type:", m\_OurLandscape->EventtypeToString( event ) );

exit( 1 );

}

if ( m\_CurrentSkState == toss\_FDying ) return true; else

return false;

}

|  |  |  |  |  |  |
| --- | --- | --- | --- | --- | --- |
| void Skylark\_Female::OnMaleNeverComesBack | ( | Skylark\_Male \* | *AMale* | ) |  |

References Skylark\_Adult::BSuccess, MyMate, and Skylark\_Adult::Paired.

Referenced by Skylark\_Male::OnPairing(), and Skylark\_Male::st\_Immigrating().

{

#ifdef \_\_CJTDebug\_5

if ( IsAlive() != 0xDEADC0DE )

DEADCODEError();

#endif

if ( MyMate == AMale ) {

MyMate = NULL;

Paired = false;

BSuccess = false;

} //This could cause problems if this function is called late

}

|  |  |  |  |  |
| --- | --- | --- | --- | --- |
| void Skylark\_Female::OnMateDying | ( |  | ) |  |

References g\_stopdate, Skylark\_Base::m\_CurrentSkState, TAnimal::m\_OurLandscape, Skylark\_Base::m\_OurPopulationManager, Skylark\_Base::m\_OurTerritories, MyClutch, MyMate, Skylark\_Adult::MyTerritory, Skylark\_Clutch::OnMumGone(), skTerritory\_struct::ref, SkTerritories::RemoveFemale(), Landscape::SupplyDayInYear(), toss\_FFloating, toss\_FFlocking, and Skylark\_Population\_Manager::WriteSKPOM1().

Referenced by Skylark\_Population\_Manager::Catastrophe(), and Skylark\_Male::st\_Dying().

{

#ifdef \_\_CJTDebug\_5

if ( IsAlive() != 0xDEADC0DE )

DEADCODEError();

#endif

// Assumes that she forgets about any active brood

MyMate = NULL;

if ( MyClutch ) {

MyClutch->OnMumGone();

MyClutch = 0;

}

if ( MyTerritory.ref != -1 ) {

m\_OurTerritories->RemoveFemale( MyTerritory.ref );

MyTerritory.ref = -1;

#ifdef \_\_SKPOM

m\_OurPopulationManager->WriteSKPOM1( m\_OurLandscape->SupplyDayInYear(), 1006 );

#endif

}

if ( m\_OurLandscape->SupplyDayInYear() > g\_stopdate ) m\_CurrentSkState = toss\_FFlocking; else

m\_CurrentSkState = toss\_FFloating;

}

|  |  |  |  |  |
| --- | --- | --- | --- | --- |
| void Skylark\_Female::OnMateHomeless | ( |  | ) |  |

References Skylark\_Adult::BSuccess, g\_stopdate, Skylark\_Base::m\_CurrentSkState, TAnimal::m\_OurLandscape, Skylark\_Base::m\_OurPopulationManager, MyClutch, MyMate, Skylark\_Adult::MyTerritory, Skylark\_Clutch::OnMumGone(), Skylark\_Adult::Paired, skTerritory\_struct::ref, Landscape::SupplyDayInYear(), toss\_FFloating, toss\_FFlocking, and Skylark\_Population\_Manager::WriteSKPOM1().

Referenced by Skylark\_Male::OnReHouse().

{

#ifdef \_\_CJTDebug\_5

if ( IsAlive() != 0xDEADC0DE )

DEADCODEError();

#endif

if ( MyClutch ) {

MyClutch->OnMumGone();

MyClutch = 0;

#ifdef \_\_SKPOM

m\_OurPopulationManager->WriteSKPOM1( m\_OurLandscape->SupplyDayInYear(), 1007 );

#endif

}

MyMate = NULL;

Paired = false;

BSuccess = false;

MyTerritory.ref = -1;

if ( m\_OurLandscape->SupplyDayInYear() > g\_stopdate ) m\_CurrentSkState = toss\_FFlocking; else

m\_CurrentSkState = toss\_FFloating;

}

|  |  |  |  |  |  |
| --- | --- | --- | --- | --- | --- |
| void Skylark\_Female::OnSetMyClutch | ( | Skylark\_Clutch \* | *p\_C* | ) |  |

inline

Referenced by Skylark\_Population\_Manager::CreateObjects().

{

MyClutch = p\_C;

}

|  |  |  |  |  |
| --- | --- | --- | --- | --- |
| void Skylark\_Female::OnStopFeedingChicks | ( |  | ) |  |

References Skylark\_Base::m\_CurrentSkState, toss\_FCaringForYoung, and toss\_StartingNewBrood.

Referenced by Skylark\_Male::OnPreFledgelingMature(), and Skylark\_Male::st\_ScaringOffChicks().

{

// Only a problem if she has to be told to stop feeding

if ( m\_CurrentSkState == toss\_FCaringForYoung )

m\_CurrentSkState = toss\_StartingNewBrood;

}

|  |  |  |  |  |
| --- | --- | --- | --- | --- |
| virtual void Skylark\_Female::PesticideResponse | ( |  | ) |  |

protectedvirtual

Reimplemented from Skylark\_Base.

Referenced by EndStep().

|  |  |  |  |  |
| --- | --- | --- | --- | --- |
| void Skylark\_Female::ResetBreedingSuccess | ( |  | ) |  |

References m\_BreedingSuccess.

Referenced by Skylark\_Female(), st\_Immigrating(), and Skylark\_Population\_Manager::TheBreedingSuccessProbe().

{

#ifdef \_\_CJTDebug\_5

if ( IsAlive() != 0xDEADC0DE )

DEADCODEError();

#endif

m\_BreedingSuccess=-1;

}

|  |  |  |  |  |
| --- | --- | --- | --- | --- |
| void Skylark\_Female::SensibleCopy | ( |  | ) |  |

|  |  |  |  |
| --- | --- | --- | --- |
| skSpiralResult Skylark\_Female::Spiral | ( | int | *x*, |
|  |  | int | *y*, |
|  |  | int | *radius* |
|  | ) |  |  |

protected

|  |  |  |  |
| --- | --- | --- | --- |
| skSpiralResult Skylark\_Female::Spiral2 | ( | int | *x*, |
|  |  | int | *y*, |
|  |  | int | *radius* |
|  | ) |  |  |

protected

|  |  |  |  |  |
| --- | --- | --- | --- | --- |
| bool Skylark\_Female::st\_Arriving | ( |  | ) |  |

protected

Ensures that the bird is not paired on arrival and checks the weather status. If bad weather then returns initiates a transition to st\_TempLeaveArea otherwise to st\_FindTerritory.

References g\_rand\_uni, Skylark\_Adult::GetBadWeather(), TAnimal::m\_Location\_x, TAnimal::m\_Location\_y, TAnimal::m\_OurLandscape, Skylark\_Adult::Paired, Landscape::SupplySimAreaHeight(), and Landscape::SupplySimAreaWidth().

Referenced by Step().

{

#ifdef \_\_CJTDebug\_5

if ( IsAlive() != 0x0DEADC0DE ) DEADCODEError();

#endif

Paired = false;

if ( GetBadWeather() ) return false;

else

{

m\_Location\_x = (int) floor(g\_rand\_uni()\*m\_OurLandscape->SupplySimAreaHeight());

m\_Location\_y = (int) floor(g\_rand\_uni()\*m\_OurLandscape->SupplySimAreaWidth());

return true; // TransferToState(FindTerritory)

}

}

|  |  |  |  |  |
| --- | --- | --- | --- | --- |
| int Skylark\_Female::st\_BuildingUpResources | ( |  | ) |  |

protected

The female forages from her home range each day. On 1st April she will make a transition to preparing for breeding which determines the time needed for egg production and nest building.

References April, Breed\_Temp\_Thresh, EggCounter, g\_stopdate, Skylark\_Adult::GetBadWeather(), m\_MinFemaleAcceptScore, TAnimal::m\_OurLandscape, May, MyMate, Skylark\_Male::Supply\_TerritoryQual(), Landscape::SupplyDayInYear(), and Landscape::SupplyTemp().

Referenced by Step().

{

#ifdef \_\_CJTDebug\_5

if ( IsAlive() != 0x0DEADC0DE ) DEADCODEError();

#endif

int today = m\_OurLandscape->SupplyDayInYear();

if ( today >= g\_stopdate ) {

return 2; // GiveUpTerritory

}

if ( GetBadWeather() ) return 2;

if ( ( m\_OurLandscape->SupplyTemp() > Breed\_Temp\_Thresh ) )

{

double qual = MyMate->Supply\_TerritoryQual();

if ( qual < m\_MinFemaleAcceptScore ) {

// May need to give up territory or might be because the crop has not

// started growing in which case she will wait until May

if ( today >= ( May + 14 ) ) return 2; // GiveUpTerritory

}

EggCounter = 0;

if ( today > April ) return 1; // transferToState(PrepareForBreeding)

}

return 0; // just wait

}

|  |  |  |  |  |
| --- | --- | --- | --- | --- |
| int Skylark\_Female::st\_CaringForYoung | ( |  | ) |  |

protected

Calls Skylark\_Female::FeedYoung to get food and give it to the chicks. Once the chicks reach 18 days of age if more breeding is possible then the bird will start a new brood unless it is late in the season or there have been too many breeding attempts in which case she will stop breeding when the chicks are 30 days old. In previous versions there was a bad weather component, but this has been removed since it did not contribute to the POM fit. If future analysis finds a relationship it should be incorporated here.

References FeedYoung(), g\_stopdate, m\_BreedingAttempts, TAnimal::m\_OurLandscape, MyMate, NestLoc, Skylark\_Male::SupplyBroodAge(), and Landscape::SupplyDayInYear().

Referenced by BeginStep().

{

#ifdef \_\_CJTDebug\_5

if ( IsAlive() != 0x0DEADC0DE ) DEADCODEError();

#endif

FeedYoung(); // get food and give it to the young

int ch\_age = MyMate->SupplyBroodAge( 0 );

if ( ch\_age == 18 )

// Must stop feeding and start to build a nest. She is ready to

// build nest when the young are 18 days old

{

if ( ( m\_BreedingAttempts < 4 ) && ( m\_OurLandscape->SupplyDayInYear() < g\_stopdate ) )

{

NestLoc = false; // no nest location now

return 1; //Start a new brood

} else if (ch\_age == 30) return 2;

}

return 0;

}

|  |  |  |  |  |
| --- | --- | --- | --- | --- |
| void Skylark\_Female::st\_Dying | ( |  | ) |  |

protected

References TALMaSSObject::CurrentStateNo, Skylark\_Base::m\_CurrentSkState, TAnimal::m\_OurLandscape, Skylark\_Base::m\_OurPopulationManager, Skylark\_Base::m\_OurTerritories, MyClutch, MyMate, Skylark\_Male::MyMate, Skylark\_Adult::MyTerritory, Skylark\_Male::OnMateDying(), Skylark\_Clutch::OnMumGone(), skTerritory\_struct::ref, SkTerritories::RemoveFemale(), Landscape::SupplyDayInYear(), toss\_Destroy, Landscape::Warn(), and Skylark\_Population\_Manager::WriteSKPOM1().

Referenced by Step().

{

#ifdef \_\_CJTDebug\_5

if ( IsAlive() != 0x0DEADC0DE ) DEADCODEError();

#endif

if ( MyMate ) {

if ( MyMate->MyMate != this ) {

g\_land->Warn( "Skylark\_Female::st\_Dying - Debug1 ", NULL ); exit( 1 );

}

MyMate->OnMateDying();

MyMate = NULL;

}

if ( MyTerritory.ref != -1 ) {

m\_OurTerritories->RemoveFemale( MyTerritory.ref );

MyTerritory.ref = -1;

}

if ( MyClutch ) {

MyClutch->OnMumGone();

MyClutch = NULL;

#ifdef \_\_SKPOM

m\_OurPopulationManager->WriteSKPOM1( m\_OurLandscape->SupplyDayInYear(), 1005 );

#endif

}

m\_CurrentSkState = toss\_Destroy; // will kill it at end of step!

CurrentStateNo = -1;

}

|  |  |  |  |  |
| --- | --- | --- | --- | --- |
| int Skylark\_Female::st\_EggHatching | ( |  | ) |  |

protected

A transition is made to Care for Young.

References m\_toowet.

Referenced by Step().

{

#ifdef \_\_CJTDebug\_5

if ( IsAlive() != 0x0DEADC0DE ) DEADCODEError();

#endif

m\_toowet = 0;

return 1; // TransferToState(CareForYoung)

}

|  |  |  |  |  |
| --- | --- | --- | --- | --- |
| int Skylark\_Female::st\_Emigrating | ( |  | ) |  |

protected

Is called once a day. This state determines the return to breeding areas based on probabilities that alter with date. Once suitable conditions prevail there will be a transition to st\_Immigrating.

References Skylark\_Base::Age, April, February, g\_stopdate, Skylark\_Adult::GoodWeather, January, TAnimal::m\_OurLandscape, March, May, random(), Landscape::SupplyDayInYear(), and Landscape::SupplyTemp().

Referenced by Step().

{

#ifdef \_\_CJTDebug\_5

if ( IsAlive() != 0x0DEADC0DE ) DEADCODEError();

#endif

int today = m\_OurLandscape->SupplyDayInYear();

if ( today >= g\_stopdate ) return 0; //don't come back this year

if ( m\_OurLandscape->SupplyTemp() > 0.0 ) GoodWeather++; else

GoodWeather = 0;

if ( GoodWeather >= 7 ) // 7 days of good weather

{

int chance = random( 10000 ); // number between 0-9999

if ( Age < 1 ) // if first year

{

if ( ( chance < 300 ) && ( today >= March + 15 ) ) return 1;

else if ( today >= April + 15 ) return 1;

} else {

if ( ( chance < 7 ) && ( today >= January + 14 ) ) return 1;

else if ( ( chance < 33 ) && ( today >= February + 14 ) ) return 1;

else if ( ( chance < 400 ) && ( today >= March + 14 ) ) return 1;

else if ( ( chance < 1000 ) && ( today >= April + 14 ) )return 1;

else if ( today > ( May ) ) return 1;

}

}

return 0; // return value of 0 do nothing, 1 go to immigration

}

|  |  |  |  |  |
| --- | --- | --- | --- | --- |
| int Skylark\_Female::st\_Finding\_Territory | ( |  | ) |  |

protected

References Skylark\_Adult::BSuccess, EstablishTerritory(), g\_msg, Skylark\_Adult::GetBadWeather(), Skylark\_Male::HaveTerritory, TAnimal::m\_Location\_x, TAnimal::m\_Location\_y, m\_MinFemaleAcceptScore, Skylark\_Base::m\_OurTerritories, MyMate, skTerritory\_struct::nqual, Skylark\_Male::OnMateNeverComesBack(), Skylark\_Adult::Paired, SkTerritories::Supply\_F\_Owner(), SkTerritories::Supply\_Owner(), Skylark\_Male::Supply\_Territory(), SkTerritories::SupplyNoTerritories(), MapErrorMsg::Warn(), and WARN\_BUG.

Referenced by Step().

{

#ifdef \_\_CJTDebug\_5

if ( IsAlive() != 0x0DEADC0DE ) DEADCODEError();

#endif

if (GetBadWeather()) return 3;

double score = 0;

skTerritory\_struct testTerr;

// First tries to find old mate if breeding was successful

if ( ( BSuccess == true ) && ( MyMate != NULL ) ) {

// Check if mate has a territory

if ( ( MyMate->HaveTerritory == true ) && ( MyMate->Paired == false ) ) {

testTerr = MyMate->Supply\_Territory();

// \*\*CJT\*\* Changed below to EvaluateHabitatNest 10/05/04

//score = m\_OurTerritories->EvaluateHabitatNest( m\_OurTerritories->Supply\_terr( testTerr.ref ), true );

score = testTerr.nqual;

}

if ( score > m\_MinFemaleAcceptScore ) {

//Establish the territory

EstablishTerritory();

return 1; // TransferToState(BuildUpResources)

}

}

// If old-mate did not have a good territory or was not there

// then start a search for a good unoccupied territory

{

// Male't territory was not good so lie to him and say we did not

// come back

if ( MyMate )

if ( MyMate->Paired == false ) MyMate->OnMateNeverComesBack( this );

MyMate = NULL; // Forget him he is no good!

// Sorts through the list of territories until it finds a suitable one

// will remember the last 'one' until if finds a vacant really good one

int NoTerrs = m\_OurTerritories->SupplyNoTerritories();

Skylark\_Male \* A\_Male;

int remember = -1;

for ( int i = 0; i < NoTerrs; i++ ) {

A\_Male = m\_OurTerritories->Supply\_Owner( i );

// Is territory occupied?

if ( A\_Male != NULL ) {

// Check if a female is already present

if ( m\_OurTerritories->Supply\_F\_Owner( i ) == NULL ) {

#ifdef \_\_CJTDebug\_5

if ( A\_Male->Paired ) {

g\_msg->Warn( WARN\_BUG, "Skylark\_Female::st\_Finding\_Territory(): Male already paired!", "" );

exit( 1 ); // Inconsistency

}

#endif

testTerr = A\_Male->Supply\_Territory();

/\* \*\*\* CJT \*\*\* Changed 4/6/2009. Now requires the male to set the value in his m\_MyTerritory

\*\*CJT\*\* Changed below to EvaluateHabitatNest 10/05/04

score = m\_OurTerritories->EvaluateHabitatNest( m\_OurTerritories->Supply\_terr( testTerr.ref ), true );

\*/

if ( testTerr.nqual >= m\_MinFemaleAcceptScore+5 ) {

//Pair with this one

//Establish the territory

MyMate = A\_Male;

EstablishTerritory();

Paired = true;

return 1; // TransferToState(BuildUpResources)

} else if ( testTerr.nqual >= m\_MinFemaleAcceptScore ) remember = i;

}

}

}

if ( remember == -1 ) {

#ifdef FOR\_DEMONSTRATION

m\_Location\_x = 0;

m\_Location\_y = 0;

#endif

return 2; // TransferToState(Floating);

} else {

//Pair with the male remember

MyMate = m\_OurTerritories->Supply\_Owner( remember );

//Establish the territory

EstablishTerritory();

return 1; // TransferToState(BuildUpResources)

}

}

}

|  |  |  |  |  |
| --- | --- | --- | --- | --- |
| int Skylark\_Female::st\_Floating | ( |  | ) |  |

protected

References g\_stopdate, Skylark\_Adult::GetBadWeather(), TAnimal::m\_Location\_x, TAnimal::m\_Location\_y, TAnimal::m\_OurLandscape, Skylark\_Base::m\_OurPopulationManager, random(), Landscape::SupplyDayInYear(), Population\_Manager::SupplySimH(), and Population\_Manager::SupplySimW().

Referenced by BeginStep().

{

#ifdef \_\_CJTDebug\_5

if ( IsAlive() != 0x0DEADC0DE ) DEADCODEError();

#endif

if ( GetBadWeather() ) return 3;

m\_Location\_x = random( m\_OurPopulationManager->SupplySimW() );

m\_Location\_y = random( m\_OurPopulationManager->SupplySimH() );

if ( m\_OurLandscape->SupplyDayInYear() > g\_stopdate ) {

return 2;

} else {

#ifdef FOR\_DEMONSTRATION

m\_Location\_x = 0;

m\_Location\_y = 0;

#endif

return 1; //TransferToState(FindTerritory);

}

}

|  |  |  |  |  |
| --- | --- | --- | --- | --- |
| int Skylark\_Female::st\_Flocking | ( |  | ) |  |

protected

The bird is assumed to move around the area waiting until weather conditions are suitable to begin Emigration, depending upon the time of year. Only arrives here after breeding, so poor conditions will trigger a transition to Emigration. A transition to Emigration will also occur if the bird has not emigrated before October.

References Skylark\_Adult::GetBadWeather(), Skylark\_Adult::GoodWeather, TAnimal::m\_Location\_x, TAnimal::m\_Location\_y, TAnimal::m\_OurLandscape, October, September, and Landscape::SupplyDayInYear().

Referenced by Step().

{

#ifdef \_\_CJTDebug\_5

if ( IsAlive() != 0x0DEADC0DE ) DEADCODEError();

#endif

#ifdef FOR\_DEMONSTRATION

// Removes itinerant dots from the screen, but has no effect on simulation

m\_Location\_x = 0;

m\_Location\_y = 0;

#endif

if ( m\_OurLandscape->SupplyDayInYear() > October ) {

GoodWeather = 0;

return 1; //TransferToState(Emigration);

}

bool bad=GetBadWeather();

if ( (bad) && ( m\_OurLandscape->SupplyDayInYear() >= September )) return 1; //TransferToState(Emigration);

return 0; // Carry on

}

|  |  |  |  |  |
| --- | --- | --- | --- | --- |
| int Skylark\_Female::st\_GivingUpTerritory | ( |  | ) |  |

protected

References Skylark\_Adult::BSuccess, EggCounter, FeedYoung(), TAnimal::m\_OurLandscape, Skylark\_Base::m\_OurPopulationManager, Skylark\_Base::m\_OurTerritories, MyClutch, MyMate, Skylark\_Adult::MyTerritory, NestLoc, Skylark\_Male::OnMateLeaving(), Skylark\_Clutch::OnMumGone(), Skylark\_Adult::Paired, skTerritory\_struct::ref, SkTerritories::RemoveFemale(), Skylark\_Male::SupplyBroodSize(), Landscape::SupplyDayInYear(), and Skylark\_Population\_Manager::WriteSKPOM1().

Referenced by Step().

{

#ifdef \_\_CJTDebug\_5

if ( IsAlive() != 0x0DEADC0DE ) DEADCODEError();

#endif

// If she is incubating give a message to the clutch that she is gone

if ( MyClutch ) {

MyClutch->OnMumGone();

MyClutch = NULL;

#ifdef \_\_SKPOM

m\_OurPopulationManager->WriteSKPOM1( m\_OurLandscape->SupplyDayInYear(), 1008 );

#endif

}

// Will carry on feeding the young whilst they are needing fed

if ( MyMate->SupplyBroodSize() > 0 ) {

FeedYoung();

return 0; // carry on

} else {

// No young to look after so leave the territory

EggCounter = 0;

NestLoc = false;

// No longer paired

Paired = false;

// Tell the male you are leaving

MyMate->OnMateLeaving();

// if no breeding success then forget him

if ( !BSuccess )

MyMate = NULL;

// de-register the territory ownership (if she owns one)

if ( MyTerritory.ref != -1 ) m\_OurTerritories->RemoveFemale( MyTerritory.ref );

MyTerritory.ref = -1; // No territory

return 1; // TransferToState(Floating);

}

}

|  |  |  |  |  |
| --- | --- | --- | --- | --- |
| bool Skylark\_Female::st\_Immigrating | ( |  | ) |  |

protected

An instantaneous state which determines the chanve of migration mortality. If she does die then she has to inform any old mate that she has gone. If he is already paired with another bird then can just forget him. If not dying then breeding success for this year is reset and the bird transitions to st\_Arrival.

References Skylark\_Base::Age, GetMigrationMortality(), m\_BreedingAttempts, m\_MinFemaleAcceptScore, m\_pesticide\_sprayed, MyClutch, MyMate, Skylark\_Male::OnMateNeverComesBack(), Skylark\_Adult::Paired, random(), ResetBreedingSuccess(), and CfgFloat::value().

Referenced by Step().

{

#ifdef \_\_CJTDebug\_5

if ( IsAlive() != 0x0DEADC0DE ) DEADCODEError();

#endif

if ( ( random( 100 ) < GetMigrationMortality() ) || Age > 5 ) {

if ( MyMate != NULL ) {

// if she has no mate she don't care

// Otherwise he receives a bogus on Mate death message

if ( !MyMate->Paired ) {

//Not paired so he is told to forget her

MyMate->OnMateNeverComesBack( this );

}

// He is paired so don't need to tell him anything - forget him

// or have already told him so forget him anyway

MyMate = NULL;

#ifdef \_\_CJTDebug\_8

if ( MyClutch != NULL )

int rubbish = 0;

#endif

}

return false; // TransfertoState(Die)

} else {

ResetBreedingSuccess();

m\_BreedingAttempts = 0;

m\_pesticide\_sprayed = false; // reset the sprayed flag

m\_MinFemaleAcceptScore = cfg\_FemaleMinTerritoryAcceptScore.value();

return true; // TransferToState(Arrival)

}

}

|  |  |  |  |  |
| --- | --- | --- | --- | --- |
| int Skylark\_Female::st\_Incubating | ( |  | ) |  |

protected

Incubation occurs as described in development below. The female spends that time off the nest required to find energy to cover her basal metabolic requirements, plus that energy required to warm the eggs. Incubation continues until the eggs hatch and there is a transition to Care For Young, or the incubation period (MID) is exceeded, at which point the female will Start New Brood. This state can only be left by a call to OnEggHatch being altered on creation of a nestling or to StartNewBrood

References CalcFoodTime(), m\_Counter1, m\_NestTime, TAnimal::m\_OurLandscape, Skylark\_Base::m\_OurPopulationManager, m\_toowet, MaxFeedRain, Landscape::SupplyDaylength(), Skylark\_Population\_Manager::SupplyEMi(), and Landscape::SupplyRain().

Referenced by Step().

{

#ifdef \_\_CJTDebug\_5

if ( IsAlive() != 0x0DEADC0DE ) DEADCODEError();

#endif

// Must calculate time needed to feed

// will ignore time off nest due to disturbance

m\_NestTime = 0;

if ( --m\_Counter1 > 0 )

{

// ExtraBroodHeat extra because of heat for eggs

double calories\_needed = m\_OurPopulationManager->SupplyEMi();

int foodtime = CalcFoodTime( calories\_needed );

int Daylength = m\_OurLandscape->SupplyDaylength();

double rain = m\_OurLandscape->SupplyRain();

if ( rain > MaxFeedRain )

{

m\_toowet++;

}

if ( m\_toowet > 2 )

{

//m\_OurPopulationManager->WriteToTest2File( m\_OurLandscape->SupplyDayInYear(), 1003 );

return 3; //TransferToState(StartNewBrood);

} else

{

if ( foodtime > Daylength ) foodtime = Daylength;

m\_NestTime = ( 24 \* 60 - foodtime ); //add night time

return 0; // wait

}

}

//m\_OurPopulationManager->WriteToTest2File( m\_OurLandscape->SupplyDayInYear(), 1002 );

return 3; // TransferToState(StartNewBrood);

}

|  |  |  |  |  |
| --- | --- | --- | --- | --- |
| int Skylark\_Female::st\_Laying | ( |  | ) |  |

protected

References Skylark\_Clutch::AddEgg(), Skylark\_struct::bx, Skylark\_struct::by, CalculateEggNumber(), Skylark\_Population\_Manager::CreateObjects(), g\_msg, Skylark\_struct::L, m\_BreedingAttempts, m\_CIPEGrid, m\_Counter1, m\_EggNumber, TAnimal::m\_Location\_x, TAnimal::m\_Location\_y, TAnimal::m\_OurLandscape, Skylark\_Base::m\_OurPopulationManager, Skylark\_Base::m\_OurTerritories, Skylark\_struct::mh, skClutch\_struct::Mum, MyClutch, MyMate, skClutch\_struct::No, Skylark\_Clutch::StartDeveloping(), Population\_Manager::SupplySimH(), Population\_Manager::SupplySimW(), Landscape::SupplyVegType(), Skylark\_struct::Terrs, MapErrorMsg::Warn(), WARN\_BUG, Skylark\_struct::x, and Skylark\_struct::y.

Referenced by Step().

{

if ( MyMate == NULL ) {

g\_msg->Warn( WARN\_BUG, "Skylark\_Female::st\_Laying(): No mate!", "" );

exit( 1 );

}

#ifdef \_\_CJTDebug\_5

if ( IsAlive() != 0x0DEADC0DE ) DEADCODEError();

#endif

// Timing called Once each day

// lays 1 egg per day until the correct number of eggs are laid

// Counter1 must be set to -1 on first time called

if ( m\_Counter1 == -1 ) {

// If we have not calculated how many eggs we need do that

m\_Counter1 = CalculateEggNumber();

m\_EggNumber = m\_Counter1;

// For the CIPE Grid statistics we need to remember this location

if (m\_BreedingAttempts<6) {

m\_CIPEGrid[0][m\_BreedingAttempts] = m\_Location\_x;

m\_CIPEGrid[1][m\_BreedingAttempts] = m\_Location\_y;

}

// The line below counts breeding attempts as at least one egg

m\_BreedingAttempts++;

skClutch\_struct \* cs;

cs = new skClutch\_struct;

cs->L = m\_OurLandscape;

cs->Mum = this;

cs->No = 0;

cs->Terrs = m\_OurTerritories;

#ifdef FOR\_DEMONSTRATION

cs->x = m\_Location\_x + 3;

cs->y = m\_Location\_y + 3;

#else

cs->x = m\_Location\_x;

cs->y = m\_Location\_y;

#endif

cs->bx = m\_Location\_x;

cs->by = m\_Location\_y;

// This sets the veg type for ever for the life of this nest and any eggs it contains

cs->mh = m\_OurLandscape->SupplyVegType( m\_Location\_x, m\_Location\_y );

if ( cs->x >= m\_OurPopulationManager->SupplySimW() ) cs->x -= m\_OurPopulationManager->SupplySimW();

if ( cs->y >= m\_OurPopulationManager->SupplySimH() ) cs->y -= m\_OurPopulationManager->SupplySimH();

m\_OurPopulationManager->CreateObjects( 0, this, NULL, cs, 1 );

delete cs;

}

// Put an egg in the clutch

MyClutch->AddEgg();

if ( --m\_Counter1 == 0 ) {

// All eggs must be laid

m\_Counter1 = 18; // This will count how many day spent incubating

MyClutch->StartDeveloping();

return 1; //TransferToState(Incubation);

}

return 0;

}

|  |  |  |  |  |
| --- | --- | --- | --- | --- |
| int Skylark\_Female::st\_MakingNest | ( |  | ) |  |

protected

Tests for the necessary territory quality, if OK finds a nest location and builds the nest. Nest attempts are assumed to be breeding attempts.

References g\_msg, June, m\_BreedingSuccess, m\_Counter1, TAnimal::m\_Location\_x, TAnimal::m\_Location\_y, m\_MinFemaleAcceptScore, TAnimal::m\_OurLandscape, Point::m\_x, Point::m\_y, May, MyMate, NestLoc, Skylark\_Male::OnNestLocation(), Skylark\_Male::Supply\_TerritoryQual(), Landscape::SupplyDayInYear(), Skylark\_Male::SupplyNestLoc(), Skylark\_Male::SupplyNestValid(), MapErrorMsg::Warn(), and WARN\_BUG.

Referenced by Step().

{

if ( MyMate == NULL ) {

g\_msg->Warn( WARN\_BUG, "Skylark\_Female::st\_MakingNest(): No mate!", "" );

exit( 1 );

}

int today = m\_OurLandscape->SupplyDayInYear();

if ( !NestLoc ) {

// First make a test whether territory is still OK

double qual = MyMate->Supply\_TerritoryQual();

if ( qual < m\_MinFemaleAcceptScore ) {

// May need to give up territory or might be because the crop has not

// started growing in which case she will wait until May

if ( today >= ( June ) ) {

return 3; // GiveUpTerritory

}

return 0;

}

// Everything OK so find a nest location

bool valid = MyMate->SupplyNestValid();

if (valid) {

Point p = MyMate->SupplyNestLoc();

m\_Location\_x = p.m\_x;

m\_Location\_y = p.m\_y;

// Must tell the male

if ( MyMate )

MyMate->OnNestLocation( p.m\_x, p.m\_y );

} else {

// m\_OurPopulationManager->WriteToTestFile(-1,-1);

if ( today >= ( May + 14 ) ) {

return 3; // GiveUpTerritory

} else

return 0;

}

// The line below counts breeding attempts as nest building

// the alternative is to count this from one egg laid in St\_Laying()

if ( m\_BreedingSuccess == -1 ) m\_BreedingSuccess = 0; //Signal breeding was possible

//m\_BreedingAttempts++;

}

// Nest location must exist

if ( m\_Counter1-- > 0 ) return 0; else

return 1; //TransferToState(Laying);

}

|  |  |  |  |  |
| --- | --- | --- | --- | --- |
| int Skylark\_Female::st\_PreparingForBreeding | ( |  | ) |  |

protected

Builds up resources whilst waiting for good enough weather to begin nest building. Transitions to MakingNest or GiveUpTerritory (if too late in the season).

References EggCounter, g\_stopdate, GetFood(), m\_BreedingAttempts, m\_Counter1, TAnimal::m\_OurLandscape, Skylark\_Adult::RemoveEM(), Resources, Landscape::SupplyDayInYear(), Landscape::SupplyDaylength(), Landscape::SupplyGlobalDate(), Landscape::SupplySnowcover(), and Landscape::SupplyTemp().

Referenced by Step().

{

#ifdef \_\_CJTDebug\_5

if ( IsAlive() != 0x0DEADC0DE ) DEADCODEError();

#endif

// Timing: Called once per day

// EggCounter was set to Zero on leaving building up resources

// Egg counter is the refraction period for producing the

// eggs - nesbuilding time

long today = m\_OurLandscape->SupplyDayInYear();

if ( today > g\_stopdate ) return 2; // too late give up

Resources += RemoveEM( GetFood( m\_OurLandscape->SupplyDaylength() ) );

if ( EggCounter++ > 5 ) {

// if ( ( Resources < Breed\_Res\_Thresh2 ) || ( m\_OurLandscape->SupplySnowcover() ) == true ) return 0; // snow or not enough resources

if ( m\_OurLandscape->SupplySnowcover() == true ) return 0; // snow

// Check that the temp is above 5 for the last three days

today = m\_OurLandscape->SupplyGlobalDate();

double t = m\_OurLandscape->SupplyTemp( today );

if ( t < 5.0 ) return 0; // too cold

t = m\_OurLandscape->SupplyTemp( today - 1 );

if ( t < 5.0 ) return 0; // too cold

t = m\_OurLandscape->SupplyTemp( today - 2 );

if ( t < 5.0 ) return 0; // too cold

// Everything is OK so build

// takes one day less to build nest on first attempt (3 rather than 4)

if ( m\_BreedingAttempts > 0 ) m\_Counter1 = 4; else {

m\_Counter1 = 3;

}

return 1; // TransferToState(MakeNest)

}

return 0;

}

|  |  |  |  |  |
| --- | --- | --- | --- | --- |
| int Skylark\_Female::st\_StartingNewBrood | ( |  | ) |  |

protected

Any current nests or clutches are removed. The female assesses the habitat quality of the territory. If still suitable (i.e. above MTQ) she will make a transition to Make Nest, otherwise she will go to Give Up Territory.

References g\_stopdate, m\_BreedingAttempts, m\_Counter1, m\_MinFemaleAcceptScore, TAnimal::m\_OurLandscape, Skylark\_Base::m\_OurPopulationManager, m\_toowet, MyClutch, MyMate, NestLoc, Skylark\_Clutch::OnMumGone(), st\_StoppingBreeding(), Skylark\_Male::Supply\_TerritoryQual(), Landscape::SupplyDayInYear(), and Skylark\_Population\_Manager::WriteSKPOM1().

Referenced by Step().

{

#ifdef \_\_CJTDebug\_5

if ( IsAlive() != 0x0DEADC0DE ) DEADCODEError();

#endif

NestLoc = false;

if ( MyClutch ) {

MyClutch->OnMumGone();

MyClutch = NULL;

#ifdef \_\_SKPOM

m\_OurPopulationManager->WriteSKPOM1( m\_OurLandscape->SupplyDayInYear(), 1009 );

#endif

}

double qual = MyMate->Supply\_TerritoryQual();

if ( qual < m\_MinFemaleAcceptScore ) {

return 2; // transferToState(GiveUpTerritory)

}

if ( ( m\_BreedingAttempts < 4 ) && ( m\_OurLandscape->SupplyDayInYear() < g\_stopdate ) ) { // Was July-4

if ( m\_BreedingAttempts > 0 ) m\_Counter1 = 4; else

m\_Counter1 = 3;

m\_toowet = 0;

return 1; // TransferToState(MakeNest)

} else {

st\_StoppingBreeding();

return 3; // Transfer to state flocking

}

}

|  |  |  |  |  |
| --- | --- | --- | --- | --- |
| int Skylark\_Female::st\_StoppingBreeding | ( |  | ) |  |

protected

Called when the female stops breeding for the year. Removes the pair bond and tell the male she is leaving. She deregisters her territory ownership and if there has been no breeding success then she forgets the male.

References Skylark\_Adult::BSuccess, TAnimal::m\_OurLandscape, Skylark\_Base::m\_OurPopulationManager, Skylark\_Base::m\_OurTerritories, MyClutch, MyMate, Skylark\_Adult::MyTerritory, Skylark\_Male::OnMateLeaving(), Skylark\_Clutch::OnMumGone(), Skylark\_Adult::Paired, skTerritory\_struct::ref, SkTerritories::RemoveFemale(), Landscape::SupplyDayInYear(), and Skylark\_Population\_Manager::WriteSKPOM1().

Referenced by st\_StartingNewBrood(), and Step().

{

#ifdef \_\_CJTDebug\_5

if ( IsAlive() != 0x0DEADC0DE ) DEADCODEError();

#endif

if ( MyClutch ) {

MyClutch->OnMumGone();

MyClutch = NULL;

#ifdef \_\_SKPOM

m\_OurPopulationManager->WriteSKPOM1( m\_OurLandscape->SupplyDayInYear(), 1010 );

#endif

}

// No longer paired

Paired = false;

// Tell the male you are leaving

MyMate->OnMateLeaving();

// if no breeding success then forget him

if ( !BSuccess )

MyMate = NULL;

// de-register the territory ownership

m\_OurTerritories->RemoveFemale( MyTerritory.ref );

MyTerritory.ref = -1; // No territory

// TransferToState(Flocking)

return 3;

}

|  |  |  |  |  |
| --- | --- | --- | --- | --- |
| int Skylark\_Female::st\_TempLeavingArea | ( |  | ) |  |

protected

Waits for one week of weather not classified as bad by Skylark\_Adult::GetBadWeather

References Skylark\_Adult::GetBadWeather(), and Skylark\_Adult::GoodWeather.

Referenced by Step().

{

#ifdef \_\_CJTDebug\_5

if ( IsAlive() != 0x0DEADC0DE ) DEADCODEError();

#endif

// Timing - called once per day

if ( !GetBadWeather() ) GoodWeather++;

else GoodWeather = 0;

if ( GoodWeather >= 7 ) // Week of not terrible weather

{

return 1; // comeback to the area

}

return 0; // return value of 0 do nothing, 1 go to Arrival

}

|  |  |  |  |  |  |
| --- | --- | --- | --- | --- | --- |
| void Skylark\_Female::Step | ( | void |  | ) |  |

virtual

Reimplemented from TAnimal.

References Calendar::Date(), g\_date, m\_Counter1, Skylark\_Base::m\_CurrentSkState, TAnimal::m\_Location\_x, TAnimal::m\_Location\_y, TAnimal::m\_OurLandscape, Skylark\_Base::m\_OurPopulationManager, m\_toowet, Skylark\_Clutch::Mother, MyClutch, NestLoc, Skylark\_Clutch::OnMumGone(), Skylark\_Adult::Paired, st\_Arriving(), st\_BuildingUpResources(), st\_Dying(), st\_EggHatching(), st\_Emigrating(), st\_Finding\_Territory(), st\_Flocking(), st\_GivingUpTerritory(), st\_Immigrating(), st\_Incubating(), st\_Laying(), st\_MakingNest(), st\_PreparingForBreeding(), st\_StartingNewBrood(), st\_StoppingBreeding(), st\_TempLeavingArea(), TALMaSSObject::StepDone, Landscape::SupplyDayInYear(), Population\_Manager::SupplyStateNames(), toss\_BuildingUpResources, toss\_Destroy, toss\_EggHatching, toss\_FArriving, toss\_FCaringForYoung, toss\_FDying, toss\_FEmigrating, toss\_FFindingTerritory, toss\_FFloating, toss\_FFlocking, toss\_FImmigrating, toss\_FTempLeavingArea, toss\_GivingUpTerritory, toss\_Incubating, toss\_Initiation, toss\_Laying, toss\_MakingNest, toss\_PreparingForBreeding, toss\_StartingNewBrood, toss\_StoppingBreeding, Landscape::Warn(), and Skylark\_Population\_Manager::WriteSKPOM1().

{

#ifdef \_\_CJTDebug\_5

if ( IsAlive() != 0x0DEADC0DE ) DEADCODEError();

#endif

if ( StepDone || m\_CurrentSkState == toss\_Destroy ) return;

#ifdef \_\_CJTDebug\_5

if ( MyClutch ) {

if ( MyClutch->Mother != this ) {

g\_land->Warn( "Skylark\_Female::Step: DeBug5 ", NULL ); exit( 1 );

}

}

#endif

switch ( m\_CurrentSkState ) {

case toss\_Initiation: // Initial

m\_CurrentSkState = toss\_FFlocking;

break;

case toss\_FFlocking: // Flocking

switch ( st\_Flocking() ) {

case 1:

// use 0,0 as a default location

m\_Location\_x = 0;

m\_Location\_y = 0;

m\_CurrentSkState = toss\_FEmigrating; // -> Emigration

break;

}

StepDone = true;

break;

case toss\_FFloating: // Floating

StepDone = true;

break;

case toss\_FArriving: // Arriving - instant

if ( st\_Arriving() ) m\_CurrentSkState = toss\_FFindingTerritory; // -> find terr.

else {

m\_CurrentSkState = toss\_FTempLeavingArea;

}

StepDone = true;

break;

case toss\_FImmigrating: // Immigration - instant

if ( st\_Immigrating() ) m\_CurrentSkState = toss\_FArriving; // -> arrival

else {

#ifdef TEST\_ISSUE\_DEATH\_WARRANT

printf( "Skylark\_Female::Step() : st\_Immigrating() : %d\n", ( int )g\_date->Date() );

#endif

m\_CurrentSkState = toss\_FDying; // -> die

}

break;

case toss\_FEmigrating: // Emigration

if ( st\_Emigrating() == 1 ) m\_CurrentSkState = toss\_FImmigrating; // -> immigr.

StepDone = true;

break;

case toss\_FTempLeavingArea: // temp.leave.area

if ( st\_TempLeavingArea() ) m\_CurrentSkState = toss\_FArriving; // -> arrival

StepDone = true;

break;

case toss\_FFindingTerritory: // FindingTerritory

switch ( st\_Finding\_Territory() ) {

case 1:

m\_CurrentSkState = toss\_BuildingUpResources; // -> building res.

#ifdef \_\_CJTDebug\_5

if ( !Paired ) {

g\_land->Warn( "Skylark\_Female::Step: !Paired ", NULL ); exit( 1 );

}

#endif

break;

case 2:

// Should never be paired here

m\_CurrentSkState = toss\_FFloating; // -> floating

break;

case 3:

m\_CurrentSkState = toss\_FTempLeavingArea;

break;

}

StepDone = true; // will now go one to EndStep

break;

case toss\_BuildingUpResources: // Building Resources

#ifdef \_\_CJTDebug\_5

if ( !Paired ) {

g\_land->Warn( "Skylark\_Female::Step: !Paired ", NULL ); exit( 1 );

}

#endif

switch ( st\_BuildingUpResources() ) {

case 1:

m\_CurrentSkState = toss\_PreparingForBreeding; // -> prepare for breed.

break;

case 2:

// m\_OurPopulationManager->WriteToTestFile(-2,-1);

m\_CurrentSkState = toss\_GivingUpTerritory; // -> give up territory

break;

}

StepDone = true;

break;

case toss\_MakingNest: // Making nest

switch ( st\_MakingNest() ) {

case 1:

m\_CurrentSkState = toss\_Laying; // -> laying

m\_Counter1 = -1; // Code for deciding many days before laying starts

break;

case 2: {

g\_land->Warn( "Skylark\_Female::Step: Illegal result ", NULL ); exit( 1 );

}

break;

case 3:

m\_CurrentSkState = toss\_GivingUpTerritory; // -> give up territory

break;

}

StepDone = true; // will now go one to EndStep

break;

case toss\_PreparingForBreeding: // PreparingForBreeding

#ifdef \_\_CJTDebug\_5

if ( !Paired ) {

g\_land->Warn( "Skylark\_Female::Step: !Paired ", NULL ); exit( 1 );

}

#endif

switch ( st\_PreparingForBreeding() ) {

case 0: // Do nothing

break;

case 1:

m\_CurrentSkState = toss\_MakingNest; // -> make nest

NestLoc = false; // no nest location yet

break;

case 2: // Too late -> give up for this year

m\_CurrentSkState = toss\_GivingUpTerritory;

break;

}

StepDone = true; // will now go one to EndStep

break;

case toss\_Laying: // Laying

if ( st\_Laying() ) {

m\_CurrentSkState = toss\_Incubating; // -> incubation

m\_toowet = 0;

}

StepDone = true;

break;

case toss\_StartingNewBrood: // StartingNewBrood

switch ( st\_StartingNewBrood() ) {

case 1:

m\_CurrentSkState = toss\_MakingNest; // -> make nest

break;

case 2:

// m\_OurPopulationManager->WriteToTestFile(-3,-1);

m\_CurrentSkState = toss\_GivingUpTerritory; // -> give up terr.

break;

case 3:

m\_CurrentSkState = toss\_FFlocking; // -> flocking

break;

}

StepDone = true; // will now go one to EndStep

break;

case toss\_EggHatching: // EggHatching - happens after the young hatch at 1 min to midnight

if ( st\_EggHatching() ) m\_CurrentSkState = toss\_FCaringForYoung; // -> care for young

StepDone = true;

break;

case toss\_Incubating: // Incubation

switch ( st\_Incubating() ) {

case 1: {

g\_land->Warn( "Skylark\_Female::Step: EggHatching ", NULL ); exit( 1 );

}

//m\_CurrentSkState=35; // -> egg hatch

break;

case 2:

g\_land->Warn( "Skylark\_Female::Step: Illegal Return Value ", NULL );

exit( 1 );

case 3:

m\_CurrentSkState = toss\_StartingNewBrood; // -> start new brood

MyClutch->OnMumGone();

MyClutch = NULL;

#ifdef \_\_SKPOM

m\_OurPopulationManager->WriteSKPOM1( m\_OurLandscape->SupplyDayInYear(), 1004 );

#endif

break;

}

StepDone = true; // will now go one to EndStep

break;

case toss\_StoppingBreeding: // StopBreed. - instant

if ( st\_StoppingBreeding() ) {

m\_CurrentSkState = toss\_FFlocking; // -> flocking

}

break;

case toss\_FDying: // Dying

st\_Dying();

StepDone = true;

break;

case toss\_GivingUpTerritory: // giving up terr. - one day

if ( st\_GivingUpTerritory() == 1 ) m\_CurrentSkState = toss\_FFloating; // -> floating

StepDone = true;

break;

default:

string st = m\_OurPopulationManager->SupplyStateNames( ( int )m\_CurrentSkState );

g\_land->Warn( "Skylark\_Female::Step: Unknown State", st.c\_str() );

exit( 1 );

}

}

|  |  |  |  |  |
| --- | --- | --- | --- | --- |
| int Skylark\_Female::Supply\_BreedingAttempts | ( |  | ) |  |

References m\_BreedingAttempts.

Referenced by Skylark\_Population\_Manager::TheBreedingSuccessProbe().

{

#ifdef \_\_CJTDebug\_5

if ( IsAlive() != 0xDEADC0DE )

DEADCODEError();

#endif

return m\_BreedingAttempts;

}

|  |  |  |  |  |
| --- | --- | --- | --- | --- |
| int Skylark\_Female::Supply\_BreedingSuccess | ( |  | ) |  |

References m\_BreedingSuccess.

Referenced by Skylark\_Population\_Manager::TheBreedingSuccessProbe().

{

#ifdef \_\_CJTDebug\_5

if ( IsAlive() != 0xDEADC0DE )

DEADCODEError();

#endif

return m\_BreedingSuccess;

}

|  |  |  |  |  |
| --- | --- | --- | --- | --- |
| int Skylark\_Female::Supply\_NestTime | ( |  | ) |  |

inline

Referenced by Skylark\_Clutch::st\_Developing().

{

return m\_NestTime;

}

|  |  |  |  |  |
| --- | --- | --- | --- | --- |
| Skylark\_Clutch\* Skylark\_Female::SupplyMyClutch | ( |  | ) |  |

inline

Referenced by Skylark\_Clutch::Step().

{

return MyClutch;

}

---

## Member Data Documentation

int Skylark\_Female::EggCounter

protected

Referenced by st\_BuildingUpResources(), st\_GivingUpTerritory(), and st\_PreparingForBreeding().

int Skylark\_Female::m\_BreedingAttempts

protected

Referenced by Skylark\_Female(), st\_CaringForYoung(), st\_Immigrating(), st\_Laying(), st\_PreparingForBreeding(), st\_StartingNewBrood(), and Supply\_BreedingAttempts().

int Skylark\_Female::m\_BreedingSuccess

protected

Referenced by ResetBreedingSuccess(), st\_MakingNest(), and Supply\_BreedingSuccess().

int Skylark\_Female::m\_CIPEGrid[2][6]

Referenced by st\_Laying().

int Skylark\_Female::m\_Counter1

protected

Referenced by OnBroodDeath(), OnClutchDeath(), st\_Incubating(), st\_Laying(), st\_MakingNest(), st\_PreparingForBreeding(), st\_StartingNewBrood(), and Step().

int Skylark\_Female::m\_EggNumber

protected

Referenced by st\_Laying().

double Skylark\_Female::m\_MinFemaleAcceptScore

protected

Referenced by BeginStep(), Skylark\_Female(), st\_BuildingUpResources(), st\_Finding\_Territory(), st\_Immigrating(), st\_MakingNest(), and st\_StartingNewBrood().

int Skylark\_Female::m\_NestTime

protected

Referenced by FeedYoung(), and st\_Incubating().

bool Skylark\_Female::m\_pesticide\_sprayed

protected

Referenced by OnFarmEvent(), Skylark\_Female(), and st\_Immigrating().

bool Skylark\_Female::m\_pesticide\_sprayed\_die

protected

int Skylark\_Female::m\_toowet

protected

Referenced by st\_EggHatching(), st\_Incubating(), st\_StartingNewBrood(), and Step().

Skylark\_Clutch\* Skylark\_Female::MyClutch

protected

Referenced by OnBroodDeath(), OnClutchDeath(), OnEggsHatch(), OnMateDying(), OnMateHomeless(), Skylark\_Female(), st\_Dying(), st\_GivingUpTerritory(), st\_Immigrating(), st\_Laying(), st\_StartingNewBrood(), st\_StoppingBreeding(), and Step().

Skylark\_Male\* Skylark\_Female::MyMate

Referenced by Skylark\_Population\_Manager::Catastrophe(), EstablishTerritory(), FeedYoung(), OnEggsHatch(), OnMaleNeverComesBack(), OnMateDying(), OnMateHomeless(), Skylark\_Female(), st\_BuildingUpResources(), st\_CaringForYoung(), st\_Dying(), Skylark\_Male::st\_Dying(), st\_Finding\_Territory(), st\_GivingUpTerritory(), Skylark\_Clutch::st\_Hatching(), st\_Immigrating(), st\_Laying(), st\_MakingNest(), st\_StartingNewBrood(), and st\_StoppingBreeding().

bool Skylark\_Female::NestLoc

protected

Referenced by st\_CaringForYoung(), st\_GivingUpTerritory(), st\_MakingNest(), st\_StartingNewBrood(), and Step().

double Skylark\_Female::Resources

protected

Referenced by EstablishTerritory(), and st\_PreparingForBreeding().

---

The documentation for this class was generated from the following files:

- skylarks\_all.h
- skylarks\_all.cpp


- Skylark\_Female
- Generated on Thu Jan 10 2013 13:15:37 for ALMaSS Skylark ODdox by
   1.8.1.1
